# A primer on the use of computational modelling to investigate affective states, affective disorders and animal welfare in non-human animals

**DOI:** 10.3758/s13415-023-01137-w

**Published:** 2023-11-30

**Authors:** Vikki Neville, Michael Mendl, Elizabeth S. Paul, Peggy Seriès, Peter Dayan

**Affiliations:** 1 Bristol Veterinary School, University of Bristol, Langford, UK; 2 Institute for Adaptive and Neural Computation, University of Edinburgh, Edinburgh, UK; 3 Max Planck Institute for Biological Cybernetics & University of Tübingen, Tübingen, Germany

## Abstract

Objective measures of animal emotion-like and mood-like states are essential for preclinical studies of affective disorders and for assessing the welfare of laboratory and other animals. However, the development and validation of measures of these affective states poses a challenge partly because the relationships between affect and its behavioural, physiological and cognitive signatures are complex. Here, we suggest that the crisp characterisations offered by computational modelling of the underlying, but unobservable, processes that mediate these signatures should provide better insights. Although this computational psychiatry approach has been widely used in human research in both health and disease, translational computational psychiatry studies remain few and far between. We explain how building computational models with data from animal studies could play a pivotal role in furthering our understanding of the aetiology of affective disorders, associated affective states and the likely underlying cognitive processes involved. We end by outlining the basic steps involved in a simple computational analysis.

## Introduction

Accurate and reliable measures of animal affect (mood-like and emotion-like states) are essential in fields such as pharmacology and neuroscience that rely on valid animal models for translation. They are also critical in animal welfare science where animal affect is increasingly viewed as the key determinant of welfare. However, even while remaining agnostic as to which animal species consciously experiences affective states (Paul et al., 2020), measuring affect in animals is challenging because it is a latent construct that can only be inferred from its multifarious components spanning behaviour, cognition and (neuro)physiology (Kremer et al., 2020; Mendl et al., 2010; Mendl & Paul, 2020). In the context of animal models relevant to affective disorders, examples include reduced sucrose consumption/preference as a marker of depression-like states (Slattery et al., 2007; Willner et al., 1987), changes in locomotor activity and willingness to enter central areas in an open field as a measure of anxiety-like states (Kumar et al., 2013; Royce, 1977), and behavioural or HPA axis responses to conditioned aversive stimuli as markers of fear- or anxiety-like states (Kumar et al., 2013). Statistical analyses are used to describe differences in these measures between control and treatment groups that receive a particular affect manipulation (e.g. chronic mild stress; Willner, 2017)) or are exposed to different housing or husbandry conditions or selectively bred for expression of particular characteristics or diseases (e.g. the Flinders sensitive line rat; Overstreet et al., 2005), or to describe linear relationships between putative affective states and the outcome measure in question.

However, translational relevance is beset with uncertainty – the links between objective measurements and subjective affect may be mediated by more than one underlying process, be influenced by other aspects of the dynamic situation, or be non-linear. As a case in point, increased locomotor activity in an open field test has been interpreted as reflecting both slower habituation to a novel environment, thus indicating *increased* anxiety (Brenes et al., 2009; Schrijver et al., 2002), or a greater willingness to explore a novel environment, thus indicating *reduced* anxiety (Carli et al., 1989). Attempts to validate behavioural and cognitive measures have had varying success, sometimes revealing a large degree of heterogeneity in the relationship between markers and the putative affective state that they are meant to reflect (Borsini et al., 2002; Forbes et al., 1996; Lagisz, Zidar, Nakagawa, Neville, Sorato, Paul, ... & Løvlie, 2020; Neville, Nakagawa, Zidar, Paul, Lagisz, Bateson, ... & Mendl, 2020b; Paul et al., 2005; Rupniak, 2003). This makes appropriate translational exploitation hard.

One important reason for this heterogeneity is that the processes and representations underpinning the latent affective states are themselves hidden, are influenced differentially by specific manipulations intended to influence affective state as a whole (Mendl et al., 2009; Mendl & Paul, 2020; Neville, Nakagawa, Zidar, Paul, Lagisz, Bateson, ... & Mendl, 2020b) and can exhibit significant individual differences. For example, animals may vary in their speed of movement, distractibility, impulsivity and the extent to which they find sucrose appetitive and electric shocks aversive. Each of these factors may potentially be influenced by affective state. However, an observed measure such as a decision/proclivity for a particular behaviour is typically a single output dimension which integrates, and thus mixes, all these variables.

The promise of computational modelling is its collection of sophisticated methods for unmixing. It does this by offering a formal and explicit description of how complex structures that are not directly observable in data, intermediate between what the animal experiences and what it does. Computational methods should thus help us understand the determinants and effects of affective states. Here, we introduce the computational analysis of behavioural data and discuss how it may be used to investigate preclinical aspects of mood disorders in non-human animals and, more generally, understand affective processes. We start by explaining the background to, and core features of, computational modelling, particularly the types of models most relevant to affect. We then discuss computational modelling from a more practical perspective; outlining how it has been used to study affective disorders in humans, and how it has been, and could be, applied to animal data.

## What is computational modelling and why is it useful?

Modelling in psychology and neuroscience has various facets. These include:

- Data analysis: quantitative ways of analysing data (e.g. using machine learning techniques to classify images or videos; Dolensek et al. , 2020; Valletta et al. , 2017).
- Mathematical modelling: mathematically operationalized descriptions of systems using layers of mechanistic and descriptive accounts (e.g. portraying the firing of neurons using the Hodgkin–Huxley model, which involves a sophisticated membrane gating process; Hodgkin & Huxley, 1952).
- Information processing (IP) modelling: characterizations of the brain as solving specifically computational problems such as maximizing reward (e.g. using a reinforcement learning framework, Sutton & Barto, 2018, to investigate how an individual might make decisions to maximise food intake).

One impressive example of the use of data analysis for animal affect is Dolensek et al. (2020)’s classifiers, which predict the emotional state of mice from images of their faces. These authors first considered how each pixel in each video frame of a mouse experiencing different treatments (some intended to generate positive valence, e.g. receiving sucrose; some generating negative valence; e.g. receiving a tailshock) differed from baseline, and they then used a machine learning approach (see Valletta et al., 2017) to distinguish between facial expressions elicited by these different treatments. They also provided extensive functional validation of their classifications. Although this sophisticated form of data analysis provided useful insights into the neuronal correlates of facial expressions in response to emotionally salient events, the classifier was purely descriptive, rather than being based on an explanation of how, for instance, the insula cortex represented emotional states and generated facial expression. This is what is often referred to as a data-driven approach: machine learning techniques are applied to large amounts of data with the aim of classification (Huys et al., 2016).

By contrast, the overarching goal of mathematical and IP modelling (which we will sometimes refer to collectively as computational modelling, to distinguish them from data analysis) is to understand data using a formal characterization of how those data might have arisen. IP modelling in particular starts from a functional view of the brain as receiving, manipulating and acting on the basis of information (Churchland & Sejnowski, 2016; Dayan, 1994), often in a surprisingly efficient manner. Here, there are explicit hypotheses about the computational mechanisms underlying the data; it is a theory-based approach (Huys et al., 2016).

For computational modelling, the ability to reproduce and predict data is a critical test of their competence. Accordingly, a core feature of such modelling is that it is generative and not just descriptive; that is, a computational model can, from suitable inputs, generate data to be compared with the results of experiments (e.g. from a decision-making task). This approach of specifying the mapping between input and output has been widely used in the field of computational psychiatry, and is particularly useful when we regard affect as a latent construct. That is, the processes characterized include the hidden structures that link affective states to the various manipulations and measurements mentioned above – i.e. the observable input and output. This underlies the power and promise of computational modelling for unearthing the neural and cognitive substrates of normative and dysfunctional affect. Computational modelling allows great specificity and clarity in the communication and testing of hypotheses and theories regarding the processes underlying the observed data. Equivalently, it provides a means for the empirical investigation of hypotheses about the cognitive and/or neural mechanisms underlying behaviour. Furthermore, the generative nature of computational modelling can drive the creation of new hypotheses – for example, one could ask what would be the behavioural output of adjusting certain parameters in the model, or considering additional modelling mechanisms. Thus, computational modelling enhances research in several regards.

Here we focus on two types of questions:

1. What is the best structural account (sometimes, model class) for the data, such as those collected in a decision-making task;
2. What are the values of the fittable parameters (sometimes called ’free’ parameters, i.e. those whose precise values are not predefined in the model) within each structural account that best capture the data (see Daw et al. , 2011; Wilson and Collins , 2019).

These questions can be asked about whole populations of subjects, treatment (or other sorts of sub-) groups, or even individuals – noting that it might well be that different subjects tackle the same situation in different ways that are best captured by different models (Piray et al., 2019). Model comparison is used to examine competing structural hypotheses about the cognitive or affective processes underlying the observed data. This involves comparing how well different models can explain the data, using various techniques to avoid overly complex models which can over-fit the data (by encompassing what is noise). Model comparison is used, for example, to assess whether animals use different approaches to solve a decision-making problem depending on their putative affective state. Parameter estimation is used to find the parameter values within any potential model that best explain the observed data (or, for Bayesians, the posterior distributions of the parameters given the observed data and priors that are part of the specification of the model), and hence to summarise the data according to the key components of the model. These estimates can be compared between animals, where there are different treatment groups and hence different estimates are anticipated, or within animals, where we might anticipate changes in an individual’s affect over time.

## Computational psychiatry: applying computational methods to examine affective disorders in humans

One central facet of the rapidly growing field of computational psychiatry is the application of computational modelling to characterise and understand neuropsychiatric disorders, including affective disorders such as major depressive disorder and generalised anxiety disorder. An essential premise of computational psychiatry is that these disorders arise from or induce a deviation from normal, often approximately optimal, behaviour, and that by defining this normativity in computationally crisp terms, we can examine the specific additional suboptimalities associated with affective disorders (Huys et al., 2016; Montague et al., 2012; Moutoussis et al., 2015; Stephan & Mathys, 2014). However, it is important to note that it is unclear whether seemingly suboptimal decision-making within a behavioural task is truly flawed, as such behaviour may serve an adaptive function outside of the context of the task (e.g. Nesse (2000); Nettle and Bateson (2012)).

Computational psychiatry studies have found that affective disorders are associated with changes in several aspects of cognition and decision-making, including reward sensitivity, learning, effort cost, and reliance on ‘hardwired’ (i.e. non-learned) behavioural tendencies. For instance, individuals suffering from affective disorders might differ from the healthy in the extent to which they employ model-free (i.e. reflexive, stimulus-response based, or habit based) versus model-based (i.e. reflective, stimulus-response-outcome based, or goal directed), and Pavlovian versus instrumental (Dayan et al., 2006; Huys et al., 2015) forms of reinforcement learning (RL) to solve problems. Model-free, compared with model-based, learning models were found to better explain the behaviour of depressed humans than non-depressed humans on a task which pitted exploration against exploitation (Blanco, Otto, Maddox, Beevers, & Love, 2013). Furthermore, Huys et al. (2012) found that humans who experienced greater levels of depression (as determined by Beck’s depression inventory, Beck et al. (1961)) had a greater tendency to dismiss further evaluation of a potential strategy when presented with a large loss, even though this was counterproductive, in a task which required them to construct a model-based representation of the environment (Huys et al., 2012). Similarly, humans experiencing post-traumatic stress disorder (PTSD) and suicidal thoughts have greater difficulty overcoming Pavlovian tendencies when it is necessary to do so, such as when making an ‘approach’ action, which is typically made to obtain rewards, to avoid a punisher (to which there is an innate tendency for an ‘avoid’ response) (Millner, den Ouden, Gershman, Glenn, Kearns, Bornstein, ... & Nock, 2019; Ousdal, Huys, Mildë, Craven, Ersland, Endestad, ... & Dolan, 2018). Effort costs have also been implicated in MDD in computational studies; depressed patients have been shown to weigh the cost of expending effort more highly than non-depressed patients (Vinckier, Jaffre, Gauthier, Smajda, Abdel-Ahad, Le Bouc, ... & Plaze , 2022), and healthy participants given an antidepressant drug (escitalopram) showed a reduction in effort costs relative to a placebo group (Meyniel, Goodwin, Deakin, Klinge, MacFadyen, Milligan, ... & Gaillard, 2016).

Computational approaches have also been useful in the development and articulation of theories about how affective disorders develop and are maintained. In line with the empirical evidence presented above, it has been argued that depression reduces recall of past outcomes (or simulation of future outcomes) that are positive, and increases recall of past actions (and simulation of future actions) that are effortful – which consequently impacts estimates of the expected value (i.e. combined outcome probability, outcome value, and effort cost) of trying to obtain rewards (Bishop & Gagne, 2018). Anxiety is said to exert a similar impact on cognition, but instead in the domain of threat avoidance; it increases recall of past actions that have negative outcomes (and simulation of future negative outcomes) and decreases recall of the effort expended for those actions (Bishop & Gagne, 2018). Others have posited that how we learn about our environment, and use the resulting information to make predictions about future outcomes, is key to affective disorders. For example, poor learning about negative outcomes could lead to a vicious cycle of an individual not being able to predict, and so repeatedly being surprised by, aversive outcomes, leading to generation of negative prediction errors and an associated persistent negative mood (Eldar et al., 2016). Computational studies have demonstrated a link between prediction errors and self-reported mood: more positive prediction errors are associated with a more positive report of mood, and vice versa (Neville et al., 2021a, c; Otto & Eichstaedt, 2018; Rutledge et al., 2014). In a similar vein, it has been suggested that depression may arise when an individual has a strong belief that they inhabit a volatile world leading to poor allostatic regulation; trying to anticipate future needs is futile when there is such uncertainty (Clark et al., 2018).

However, it is important to note that the potential precision of computationally defined components is not always perfectly matched with the often-questioned imprecision of the conventional delineations of the disorders themselves (Friston et al., 2017; Stephan, Bach, Fletcher, Flint, Frank, Friston, ... & Binder, 2016). For example, using a reinforcement learning model, a meta-analysis of human behavioural data from a probabilistic reward task revealed that anhedonic forms of major depressive disorder (MDD) was associated with a reduced reward sensitivity (Huys et al., 2013). However, a separate meta-analysis which included a broader range of studies – those comparing individuals with mood and/or anxiety disorders rather than MDD specifically – did not find reliable differences in reward sensitivity (Pike & Robinson, 2022). Instead, this meta-analysis found that patients showed faster learning about punishing outcomes and slower learning about rewarding outcomes. Other studies have suggested that mood disorders do not alter learning rates per se, but rather the extent to which learning is modulated in an environment dependent manner. For example, Browning et al (2015) found that anxiety in humans is associated with a reduced ability to modulate the speed of learning in accordance with environmental volatility (Browning et al., 2015). One hope for some in computational psychiatry is that a new nosology will emerge from unpicking these seeming contradictions (Stephan, Bach, Fletcher, Flint, Frank, Friston, ... & Binder, 2016).

In sum, computational psychiatry has arguably been instrumental in furthering our understanding of human affective disorders. Computational studies have revealed key cognitive processes which might underlie the relationship between affect and altered decision-making, which are often difficult or impossible to disentangle with statistical analyses alone. In keeping with, and in some ways extending, the precepts of RDoC (Insel, Cuthbert, Garvey, Heinssen, Pine, Quinn, ... & Wang, 2010), it has also contributed to the development of new theories of affective disorders, highlighting areas of research that may be particularly valuable in future studies and informing diagnostic and prognostic tools.

**Fig. 1:**
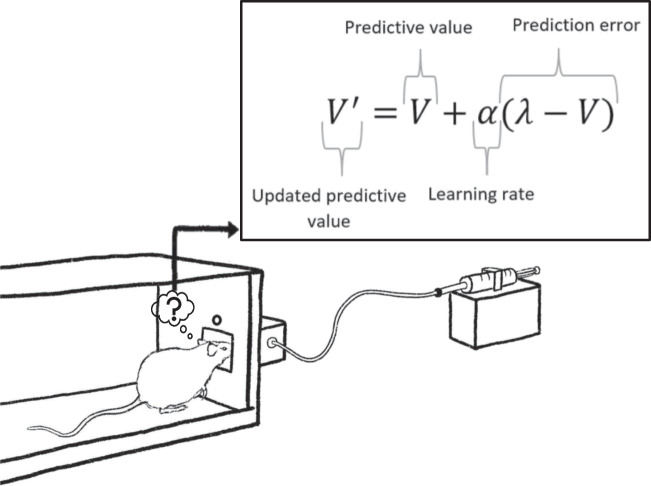
A rat in a shuttle box approaches a trough when the light above this trough is illuminated to receive a variable volume of sucrose solution. The Rescorla–Wagner learning rule can be used to learn the value of sucrose solution

## Computational approaches to study affective disorders using animal models

Computational models have been applied successfully to animal data to examine and explain the role that particular neural substrates or structures play in guiding behaviour: for instance, dopamine has been heavily implicated in the encoding of prediction errors (Glimcher, 2011; Montague et al., 1996; Schultz et al., 1993, 1997), and it has been suggested that noradrenaline could modulate behaviour by specifically altering how actions are selected (Aston-Jones & Cohen, 2005; Doya, 2002; Swanson et al., 2022). They have also been used to understand how individual differences and maladaptive behaviour might arise (Bathellier et al., 2013; Noworyta-Sokolowska et al., 2019; Rivalan et al., 2013; Spiegler et al., 2020). There is now much scope to extend these findings by examining the influence of affect on decision-making using a computational approach.

Empirical studies using computational approaches to study affective disorders inevitably differ between humans and animals. However, it is striking that computational approaches can be surprisingly similar – couched in the language of reinforcement learning (RL). Indeed, the core concepts of RL emerged from early animal behaviour studies in experimental psychology, focusing on actual or anticipated reward and punisher experience (e.g. Mendl and Paul , 2020; Rolls , 2013). While there has been much debate about what affect is (e.g. discrete vs. dimensional; Barrett et al. , 2007; Mendl et al. , 2022; Mobbs et al. , 2019) and how and whether we should semantically differentiate between affect in humans and animals (De Waal, 1999; LeDoux, 2012; Mendl et al., 2022; Panksepp, 2011), a key area of agreement across theories is that affect helps an individual to respond appropriately to both fitness-enhancing and fitness-threatening stimuli (Barrett & Finlay, 2018; LeDoux, 2012; Mendl et al., 2022; Mendl & Paul, 2020; Panksepp, 2005; Rolls, 2013), namely rewards and punishers, which are encountered by humans and non-human animals alike. At its heart, RL characterises how an individual learns to do this through interactions with the environment. i.e. how they can make decisions which maximise long-run reward intake while minimising long-run punisher exposure (Bellman, 1952; Sutton & Barto, 2018). This does not distinguish humans from other animals, and hence is an appropriate framework for translational computational modelling.

**Fig. 2:**
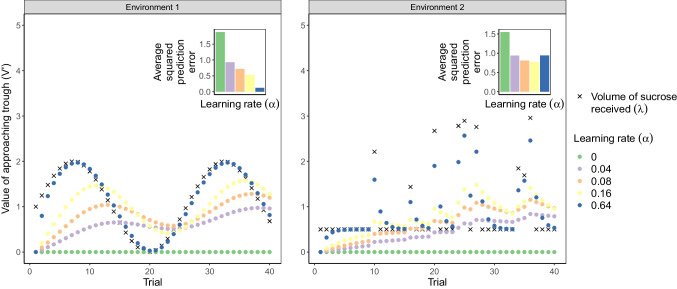
Effects of learning rate on reward tracking in different environments. The learning rate characterises the extent to which the updated value depends on the most recent outcome. The estimated value of approaching the trough, calculated using the Rescorla–Wagner learning rule shown in Fig. 1, most closely tracks the volume of sucrose solution received when the learning rate is higher; learning occurs more rapidly at higher learning rates. The *inset panels* show the average squared prediction error across trials for each learning rate in each environment. The prediction error is the difference between the reward predicted by the individual (here, the estimated value of approaching the trough) and the actual reward received on a particular trial, and so the average squared prediction captures how accurately an individual estimates the reward in the environment. In cases such as Environment 1, where the available rewards change rapidly but systematically, a high learning rate allows the individual a more accurate estimate of the likely reward value on the subsequent trial; the average squared prediction error decreases as the learning rate increases. However, in cases such as Environment 2, where the reward has a largely unchanging average, but a high variance (being delivered infrequently at random), a high learning rate may be detrimental, as the estimated value will chase the noise; the average squared prediction error is not lowest at the highest learning rate

### A reinforcement learning framework for building computational models

Building a model of decision-making within a reinforcement learning framework typically involves calculating the immediate (or long-run) value of a state, an action, or a policy to inform the actions that should be taken to maximise this value (or minimise it, in the cases of punishers). The Rescorla–Wagner learning rule (Rescorla et al., 1972; Sutton & Barto, 1981; Widrow & Hoff, 1960) is a highly popular (and influential) formal model of Pavlovian conditioning that can be applied to many situations in which an animal must learn the value of a stimulus (or action) through direct experience. It states that the change in the predictive value (*V*) of a conditioned stimulus or action following an outcome ($\lambda$) depends on the amount of signed ‘surprise’ produced by the outcome (the prediction error, which is the difference between actual and predicted outcome) and the extent to which ‘surprise’ influences learning (the learning rate, $\alpha$). More formally, the updated predictive value is the current predictive value added to a learning-rate scaled prediction error.

For example, consider a rat in a shuttle box that can visit a trough to receive a reward, such as sucrose solution, each time the light above the trough illuminates (Fig. 1.); the states could hence be ‘light illuminated’ in which the action ‘visit trough’ leads to a reward with a probability of one, and ‘light not illuminated’ in which all actions lead to a reward of zero, assuming no other source of rewards. The volume of the sucrose solution delivered, namely the magnitude of the reward, fluctuates over time such that, in order to perform optimally, the rat has to continually learn the value of approaching the trough when the light is illuminated (denoted *V* in the Rescorla–Wagner learning rule; Fig. 1). This value will depend on the experienced outcomes (denoted $\lambda$ in the Rescorla–Wagner learning rule) as well as the learning rate ($\alpha$); when learning rate is higher, the rat more closely tracks changes in experienced reward value (Fig. 2).

**Fig. 3:**
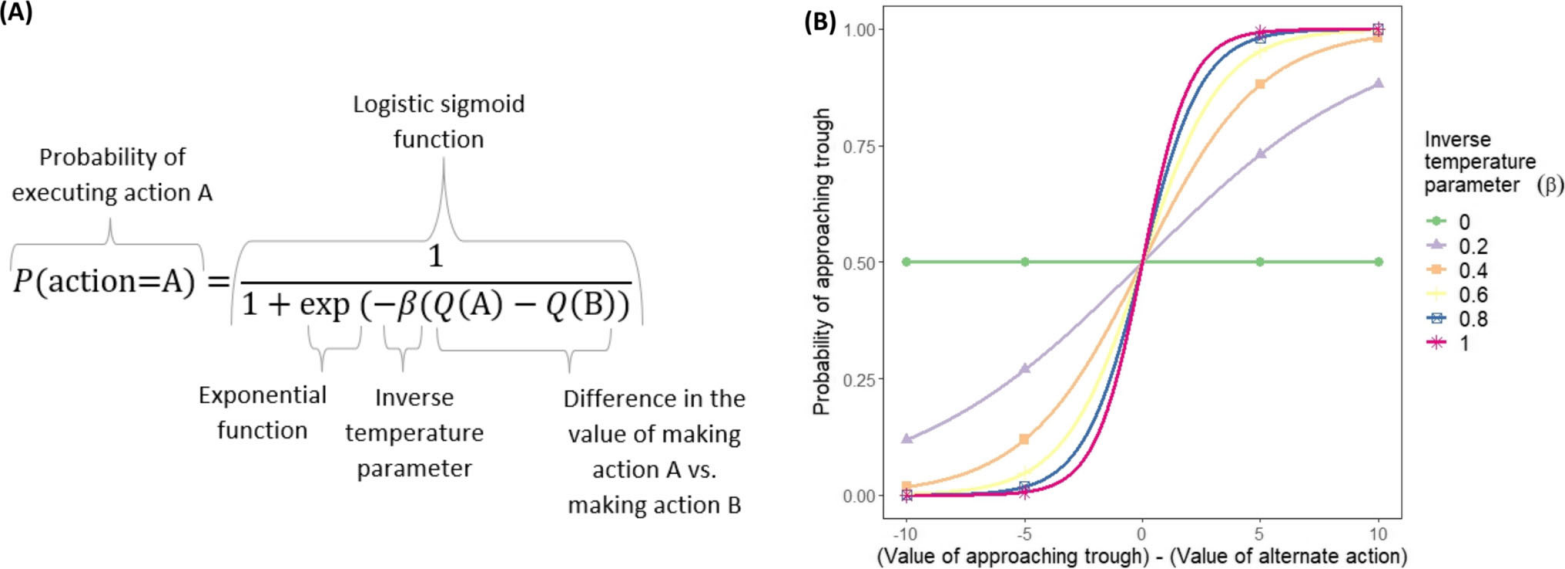
**A** The softmax function to determine the probability of executing action A when there are two possible actions (A or B) and the value of executing each action is denoted Q(A) and Q(B), respectively; **B** The softmax-determined values of the probability of approaching the trough as the difference in values between the alternate action varies, and as the inverse temperature parameter varies. Values for the inverse temperature parameter of zero would mean that the action is chosen entirely uniformly at random, while values of infinity would mean that the action with the highest value is always chosen

The rat’s decision to approach the trough following illumination of the light could depend on both the predicted value of doing so and the value of any potential alternative actions (e.g. doing nothing, or visiting an alternative food source). It is likely invalid to assume that an individual will always execute the action with the greatest value; they may indirectly make an alternate action by mistake, or may directly do so as a form of exploration that allows them to gain information about the transitions and rewards afforded by their environment. Indeed forms of matching, such as probability matching in which participants’ choices follow outcome probabilities (e.g. picking an option which offers a reward with a probability of 0.6 on just 60% of trials, even though it has the higher expected value, relative to the alternative option, on 100% of trials), are widely prevalent (Baum, 1974; Herrnstein, 1961; Vulkan, 2000). The softmax function (which is a logistic sigmoid function, a function with an S-shaped curve that maps any real value to the range 0–1, when there are only two options) provides a means to turn values of competing actions (e.g. A, which could be the predicted value of approaching the trough, and B, which could be the value of an alternative food source) into probabilities, according to inverse temperature parameter $\beta$ (Fig. 3) which specifies the randomness of choice with respect to current information. A high inverse temperature results in choices closely reflecting the relative values of competing actions, whilst choices become more random with low inverse temperatures.

This simple model illustrates that a rat’s choices between two competing actions can be conceptualised in terms of how it updates the values of the actions on the basis of information from prediction errors assimilated at a specific learning rate. This information is then used more or less faithfully, according to the inverse temperature parameter, to guide choice behaviour. Whilst this formal description of value-based decision-making may seem ‘cold’ (Loewenstein, 2000) in the sense that our description has not referred to affective influences, the quantities in the Rescorla–Wagner learning model have been argued to be tightly intertwined with affective state. Firstly, in humans, the prediction error has been shown to modulate reported affect reliably: more positive prediction errors are associated with more positively valenced affective states and vice versa (Brielmann & Dayan, 2022; Neville et al., 2021b; Otto & Eichstaedt, 2018; Rutledge et al., 2014). From this, we might be able to make inferences about the sorts of situations (i.e. unexpectedly good) that might lead to positive affect in animals to refine current non-pharmacological ways to induce particular affective states. Secondly, the role of the inverse temperature parameter in moderating the extent to which decisions are based on values is highly reminiscent of the role of affect in moderating reward processing. For example, depression can be associated with anhedonia and an impaired ability to use reward values for decision-making (American Psychiatric Association, 2013; Whitton et al., 2015), and there is some evidence from computational studies that estimates of the inverse temperature parameter fitted to data from depressed humans are lower on average than those fitted to clinically healthy humans (Blanco et al., 2013; Rupprechter et al., 2018).

**Fig. 4:**
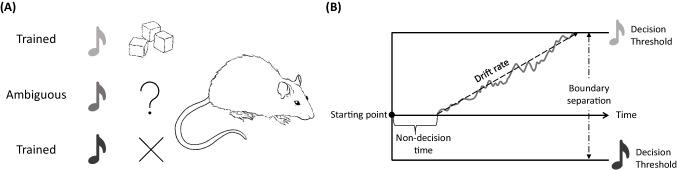
The judgement bias task and a drift-diffusion approach to modelling the ensuing data: **A** Animals are trained to associate one stimulus with a rewarding outcome, and another with an aversive outcome. The key data are how an animal responds to an ambiguous stimulus; **B** Drift-diffusion modelling describes how an animal might come to make a decision about what the ambiguous stimulus represents. The animal accumulates evidence during the stimulus presentation (*dark gray winding line*) and makes a decision when they hit a particular threshold. The time it takes to make a particular decision will depend on: where the thresholds are relative to each other (boundary separation) and to the starting point, the rate and average direction of evidence accumulation (drift rate), as well as additional non-decision time (e.g. to execute an action once a decision has been made)

### Applying computational modelling to a marker of affective state: Judgement bias

One potential measure of affect that has lent itself to computational modelling, and could be used to study affective disorders, is judgement bias (see Fig. 4a). In the characteristic paradigm quantifying this measure, subjects’ responses to stimuli that provide ambiguous information about whether an action will lead to a more- or less-preferred outcome are used to infer their affective state, with so-called ‘optimistic’ or ‘pessimistic’ resolutions of the ambiguity being associated respectively with positive and negative affect (Mendl et al., 2010; Paul et al., 2005). For example, in the first non-human task of this sort described by Harding et al. (2004), rats were trained to press a lever to obtain sucrose when one tone played, and to refrain from pressing the lever when a different tone played in order to avoid the presentation of aversive white noise. Once this discrimination was learnt, they were presented with tones intermediate between the two trained tones. When these ambiguous tones sounded, rats in putative negative affective states, resulting from unpredictable housing and husbandry conditions, were slower to press the lever and tended to be less likely to press it indicating enhanced anticipation of a negative outcome, in line with predictions.

Meta-analyses have indicated that, in general, judgement bias provides a valid measure of affective valence (Lagisz, Zidar, Nakagawa, Neville, Sorato, Paul, ... & Løvlie, 2020; Neville et al., 2021b). However, these meta-analyses also identified substantial heterogeneity in the extent to which judgement biases reflected the affective state assumed to have been induced in different studies. Insights into this heterogeneity could be offered by computational analysis approaches that can dissect how different cognitive processes underlying the decisions made (e.g. reward or punishment sensitivity / valuation; outcome probability estimation) combine to influence choices, and are affected by experimental manipulations. Computational analysis of judgement bias data is aided by the substantial framework for modelling perceptual decision-making and making decisions to maximise rewards (see Ma and Jazayeri , 2014; Ratcliff , 1978; Sutton and Barto , 2018), as well as the ease with which large amounts of data can be collected for individual animals (Jones et al., 2018).

We know of only three studies that examined judgement bias in animals using computational modelling. One of these studies employed a Bayesian decision-theoretic model to dissect the effects of short-term pre-exposure to food rewards (assumed to generate a positive state) or airpuffs (assumed to generate a relatively negative state) on subsequent decision-making under ambiguity in rats. This model begins with the reinforcement learning aligned assumption that rats will make decisions that maximise rewards and minimize punishers; they will make the ‘optimistic’ response when the expected value of doing so is great than that of the ‘pessimistic’ response. Computation of these expected values requires an estimate of the probability that the presented tone will lead to a reward (or a punisher) given a rat’s perception of the tone, the distribution of which can be calculated according to Bayes rule. This study found that rats pre-exposed to airpuffs weighted rewards more highly than punishers, in agreement with an independent measure of reward valuation. Whilst unexpected, other studies have also found that mild stress can enhance valuation of reward (Ironside et al., 2018). Also unexpectedly, these rats showed a bias towards the risk-prone response (Neville et al., 2020a). The modelling thus suggested that judgement biases do not solely arise from differences in ‘optimism’ or ‘pessimism’ in the sense of heightened or reduced expectations of the probability of rewards, but may also be influenced by variation in the context-dependent valuation of rewards or punishers. The study highlights the value of computational modelling as a tool to dissect the processes underlying affective influences on judgement biases. Whilst the original judgement bias hypothesis emphasised the role of affect in determining whether an animal probabilistically anticipates a positive or negative outcome when presented with an ambiguous stimulus, computational modelling demonstrated that this may be an oversimplification of what happens in the task, and that valuation of rewards/punishers plays a role too. In support of this, a human judgement bias study used a Bayesian decision-theoretic model to show that both perceived probability and valuation of decision outcomes may influence decision-making, and that these processes can be differentially altered by affective manipulations and even have opposing effects on decisions (Iigaya et al., 2016).

These findings indicate that computational modelling may start to provide explanations for some of the heterogeneities in judgement bias results that were revealed in recent meta-analyses. For example, two different underlying processes (e.g. estimates of outcome probability and value) mediating the interface between affect and decision-making may sometimes cancel each other out leading to a null result. It is therefore vital to understand precisely how a particular manipulation might have influenced both affect and decision-making processes, and use of computational modelling can aid this.

There exist other and more established computational models that can be applied to judgement bias data and afford valuable information about the putative processes underlying decision-making, one example of this is drift-diffusion modelling (see Fig. 4b). This is the approach that the other two studies took to investigate judgement bias in animals. These studies yielded results in line with predictions, finding that negative affect induced in rats using an anxiogenic drug increased the decision threshold for making an ‘optimistic’ response (i.e. more evidence needed to be accumulated for a decision to be made) (Hales et al., 2016), and positive affect induced using an antidepressant had drug-dependent effects on decision-making – either decreasing the evidence required to make an ‘optimistic’ versus a ‘pessimistic’ response (altered starting point), or increasing the ease with which the favourable stimuli were classified as requiring an ‘optimistic’ response (altered drift rate) (Hales et al., 2017). These models thus allowed the likely influence of affect manipulations on different putative decision-making processes to be identified.

Without these computational models, or without additional measures that more directly test whether an aspect of decision-making that could be parameterised in the model has been altered by an affect manipulation (e.g. using sucrose preference tests to measure the hedonic value of sucrose), the inferences that can be made from judgement bias studies would be restricted. For example, without drift-diffusion modelling, it would only be possible to make qualitative statements about the distribution of reaction times for the ‘optimistic’ and ‘pessimistic’ responses, rather than describe what underlies changes in the reaction time distribution precisely and quantitatively (e.g. via the values of the starting point parameter, which quantifies asymmetries in reaction time distributions for the different responses). Moreover, with standard non-computational analyses, while it is possible to infer that a particular manipulation resulted in changes in decision-making, it would be harder to infer anything about the cognitive mechanisms that may have led to the observed changes in decision-making (see Neville et al. (2020a) for example), or to assess how a combination of different factors or individual differences may influence decision-making (see Rivalan et al. (2013) for example). The Bayesian decision-theoretic modelling discussed above provides this added insight (Iigaya et al., 2016; Neville et al., 2020a). Computational models may also reveal that some of the mechanisms in the decision-making process are more tightly linked to affect than others. For example, in the case of drift-diffusion modelling, studies have shown that changes in the non-decision time parameter can reflect a whole host of factors that bear little or no relation to affect, including alcohol intoxication, age, and fatigue (Theisen et al., 2021; Ulrichsen, Alnaes, Kolskar, Richard, Sanders, Dorum, ... & Westlye, 2020; van Ravenzwaaij et al., 2012).

Computational modelling may also be more sensitive at detecting differences in affective state than conventional analyses. This is firstly because computational analyses can be based on the data as a whole, contrary to conventional analyses which typically use one-dimensional data. For example, drift-diffusion models are jointly fitted to the reaction time and accuracy data which aids identification of different types of biases, such as separating discriminability from response biases. It is secondly because, as outlined above, it allows decision-making processes that are most heavily influenced by affect to be disentangled from those that have little, no, or even opposite effects on decision-making and which may dilute the influence of affect manipulations on decision-making as a whole.

One clear example of the benefit of a computational approach comes from a judgement bias study with human subjects alluded to earlier (Iigaya et al., 2016). In this, participants completed a judgement bias task with variable rewards and losses across trials in either a pleasant room or an unpleasant room – a manipulation designed to alter affective state. No differences in decision-making between the treatment groups were identified using a conventional analysis. However, differences were observed when the data were analysed using a computational approach. Participants in the unpleasant room exhibited a stronger bias towards the ‘pessimistic’ response which would lead to more ‘pessimistic’ decision-making as predicted. At the same time, and as found in a similar animal study (Neville et al., 2020a), they weighted wins more heavily than losses (cf. Ironside et al. (2018)) which would contrarily lead to more ‘optimistic’ decision-making. The computational analysis therefore not only identified a treatment effect, with useful information about how specific processes were influenced by this treatment, but it also helped to explain why no treatment effect was observed using a conventional approach to analysis.

### Applying computational modelling in other studies of animal affect

Another decision-making task which is valuable for the study of animal affect is the probabilistic reward task. Similar to the judgement bias task, animals are first trained to discriminate between two stimuli. For example, to press a left response key when a short line appears and to press a right response key when a long line appears. Differing from the judgement bias task, testing involves assessing the extent to which a response bias emerges when asymmetries are introduced to the probabilistic schedules for the stimuli, such as rewarding correct responses to the long line with 60% probability and short line with 20% probability. Lower response biases have been reported as reflecting deficits in hedonic capacity that are characteristic of poorer mood states (e.g. anhedonic forms of major depressive disorder) (Kangas et al., 2022). The probabilistic reward task has been successfully developed for use in multiple species (Hisey et al., 2023; Kangas et al., 2020; Wooldridge et al., 2021) and it would be interesting to extend to the non-human animal versions the collection of computational modelling methods that have been applied to human data (Huys et al., 2013).

Alongside additional judgement bias studies and the probabilistic reward task, there is much scope for novel behavioural tasks to be developed for translational investigation of other affective disorders using a computational approach in animals. This would be closely aligned with the field of computational psychiatry (i.e. more direct back-translation from human to animal subjects). It will no doubt be aided by the rise in the development of accessible operant equipment (e.g. using the readily programmable Raspberry Pi), which allows increased flexibility in task design and opportunities for home-cage testing where large amounts of data can more readily be collected (Akam, Lustig, Rowland, Kapanaiah, Esteve-Agraz, Panniello, ... & Costa, 2022; Jolles, 2021).

The outcomes of such computational analyses can be examined in more detail and bolstered by investigating the neuro(physiological) underpinnings of the model. Combining computational approaches with neuronal recordings and stimulation, or with genetic models, would allow a more detailed and mechanistic understanding of the neural bases of affective disorders. Importantly, this would also allow investigation of putative causal links between neurobiological alterations and affective disorders. Opportunities for such research are obviously very limited or not possible in human participants (Saez & Gu, 2023), so non-human subjects offer far richer opportunities to advance our understanding of affect. More specifically, the influence of affect could be directly parameterized in the model, with clear hypotheses about the potential neural substrates that might be associated with these parameters. An exploratory approach could also be used; trying to identify neural substrates whose activities correlate with an aspect of the model or particular parameter, where those aspects or parameters are putatively associated with affective state. Furthermore, this linking of theory to measurable biological processes is arguably key to advancing our understanding of affective states in non-human animals. For example, this could involve exploring potential correlations between parameter estimates and physiological measures such as hippocampal atrophy (a potential neurological biomarker of MDD; (Poirier et al., 2019)), exploring how components of the model relate to real-time neural activity or physiology such as endogenous fluctuations in dopaminergic activity or serotonergic activity (both of which have been implicated in affective disorders; (Ruhé et al., 2007)), or examining how pharmacological manipulations (e.g. using antidepressants) influence parameter estimates. This would help to better elucidate the biological consequences of more positive or negative affect in a structured manner that is grounded in theory. Together with computational psychiatry research with human participants, which importantly allows insight into how affect is subjectively experienced, this will aid our understanding of the precise links between affect, behaviour and neurophysiology.

Practically, this would also allow better assessment of both the impact of potential pharmacological treatments for mood disorders and the impact of any potential refinements to animal welfare. Moreover, combined with home-cage testing, it might also be possible to repeatedly test animals and gain readouts for their affective states longitudinally. This could be highly useful for monitoring animal welfare and capturing points at which welfare is deteriorating. For instance, it could be used alongside information, such as weight loss or tumour size (Wallace, 2000), to inform humane endpoints for animal studies. This might allow greater consideration of the potential subjective experience of the animal when making these important ethical decisions.

In sum, we suggest that a computational approach will advance fields reliant on measures of animal affect by: (1) grounding research in affective theory and evidence from studies of human psychiatry; (2) paving the way for the development of computational biomarkers that provide a refined and reliable measure of affective valence; and (3) allowing a more complete and mechanistic understanding of how positive or negative affect (and good or poor welfare) is associated with changes in behavioural or (neuro)physiological markers.

## Conclusions

The field of computational psychiatry is rapidly growing and promises to further our understanding of human affective disorders. However, there is something of a dearth of translational computational psychiatry studies. This is surprising given the importance of the study of affect in non-human animals to improve our understanding of affective disorders and how to treat them (including those that we might observe in captive or companion animals), and our assessment of animal welfare. We consider that computational analyses will be able to play a pivotal role in developing improved measures of animal affect and welfare, furthering our understanding of the aetiology of affective disorders, including those that may be induced in domestic species under our management and care, and the likely underlying processes involved.

More generally, although we focused on how computational approaches can be used to capture processes of aberrant decision-making in major depressive disorder and anxiety disorders, they have also been applied to a whole host of other neuropsychiatric disorders (Series, 2020), including substance use disorders (Goldway et al., 2023; Gueguen et al., 2021), attention deficit hyperactivity disorder (ADHD) (Ging-Jehli et al., 2021), obsessive–compulsive disorder (OCD) (Fradkin et al., 2020; Loosen & Hauser, 2020), schizophrenia (Valton et al., 2017), and Tourette syndrome (Rae et al., 2019; Schüller et al., 2020). All of these could also benefit from the translational approach we are advocating.

## Data Availability

Not applicable.

## Declarations

### Open Practices Statement

There are no data/materials to be shared, and no experiments to have been preregistered.

## References

[CR1] Akam T, Lustig A, Rowland JM, Kapanaiah SK, Esteve-Agraz J, Panniello M, Márquez C, Kohl MM, Kätzel D, Costa RM (2022). Open-source, python-based, hardware and software for controlling behavioural neuroscience experiments. Elife.

[CR2] American Psychiatric Association (2013). *Diagnostic and statistical manual of mental disorders: DSM-5*. American Psychiatric Association Arlington, VA, 5th edition

[CR3] Aston-Jones G, Cohen JD (2005). Adaptive gain and the role of the locus coeruleus-norepinephrine system in optimal performance. Journal of Comparative Neurology.

[CR4] Barrett LF, Finlay BL (2018). Concepts, goals and the control of survival-related behaviors. Current Opinion in Behavioral Sciences.

[CR5] Barrett LF, Lindquist KA, Bliss-Moreau E, Duncan S, Gendron M, Mize J, Brennan L (2007). Of mice and men: Natural kinds of emotions in the mammalian brain? a response to panksepp and izard. Perspectives on Psychological Science.

[CR6] Bathellier, B., Tee, S. P., Hrovat, C., & Rumpel, S. (2013). A multiplicative reinforcement learning model capturing learning dynamics and interindividual variability in mice. *Proceedings of the National Academy of Sciences,**110*(49), 19950–19955. 10.1073/pnas.1312125110 PMC3856837 24255115

[CR7] Baum WM (1974). On two types of deviation from the matching law: Bias and undermatching 1. Journal of the Experimental Analysis of Behavior.

[CR8] Beck AT, Ward CH, Mendelson M, Mock J, Erbaugh J (1961). An inventory for measuring depression. Archives of General Psychiatry.

[CR9] Bellman R (1952). On the theory of dynamic programming. Proceedings of the National Academy of Sciences of the United States of America.

[CR10] Bishop SJ, Gagne C (2018). Anxiety, depression, and decision making: a computational perspective. Annual Review of Neuroscience.

[CR11] Blanco NJ, Otto AR, Maddox WT, Beevers CG, Love BC (2013). The influence of depression symptoms on exploratory decision-making. Cognition.

[CR12] Borsini F, Podhorna J, Marazziti D (2002). Do animal models of anxiety predict anxiolytic-like effects of antidepressants?. Psychopharmacology.

[CR13] Brenes JC, Padilla M, Fornaguera J (2009). A detailed analysis of open-field habituation and behavioral and neurochemical antidepressant-like effects in postweaning enriched rats. Behavioural brain research.

[CR14] Brielmann AA, Dayan P (2022). A computational model of aesthetic value. Psychological review.

[CR15] Browning M, Behrens TE, Jocham G, O’reilly JX, Bishop SJ (2015). Anxious individuals have difficulty learning the causal statistics of aversive environments. Nature Neuroscience.

[CR16] Carli M, Prontera C, Samanin R (1989). Effect of 5-ht1a agonists on stress-induced deficit in open field locomotor activity of rats: evidence that this model identifies anxiolytic-like activity. Neuropharmacology.

[CR17] Churchland, P. S. & Sejnowski, T. J. (2016). *The computational brain*. MIT press.

[CR18] Clark JE, Watson S, Friston KJ (2018). What is mood? a computational perspective. Psychological Medicine.

[CR19] Daw, N. D. et al. (2011). Trial-by-trial data analysis using computational models. *Decision making, affect, and learning: Attention and performance XXIII*, 23(1)

[CR20] Dayan P (1994). Computational modelling. Current Opinion in Neurobiology.

[CR21] Dayan P, Niv Y, Seymour B, Daw ND (2006). The misbehavior of value and the discipline of the will. Neural Networks.

[CR22] De Waal FB (1999). Anthropomorphism and anthropodenial: Consistency in our thinking about humans and other animals. Philosophical Topics.

[CR23] Dolensek N, Gehrlach DA, Klein AS, Gogolla N (2020). Facial expressions of emotion states and their neuronal correlates in mice. Science.

[CR24] Doya K (2002). Metalearning and neuromodulation. Neural Networks.

[CR25] Eldar E, Rutledge RB, Dolan RJ, Niv Y (2016). Mood as representation of momentum. Trends in Cognitive Sciences.

[CR26] Forbes NF, Stewart CA, Matthews K, Reid IC (1996). Chronic mild stress and sucrose consumption: Validity as a model of depression. Physiology & Behavior.

[CR27] Fradkin I, Adams RA, Parr T, Roiser JP, Huppert JD (2020). Searching for an anchor in an unpredictable world: A computational model of obsessive compulsive disorder. Psychological Review.

[CR28] Friston, K. J., Redish, A. D., & Gordon, J. A. (2017). Computational nosology and precision psychiatry. *Computational Psychiatry (Cambridge, Mass.)*, 1, 2 10.1162/CPSY_a_00001 PMC5774181 29400354

[CR29] Ging-Jehli NR, Ratcliff R, Arnold LE (2021). Improving neurocognitive testing using computational psychiatry-a systematic review for adhd. Psychological Bulletin.

[CR30] Glimcher PW (2011). Understanding dopamine and reinforcement learning: the dopamine reward prediction error hypothesis. Proceedings of the National Academy of Sciences.

[CR31] Goldway N, Eldar E, Shoval G, Hartley CA (2023). Computational mechanisms of addiction and anxiety: A developmental perspective. Biological Psychiatry.

[CR32] Gueguen MC, Schweitzer EM, Konova AB (2021). Computational theory-driven studies of reinforcement learning and decision-making in addiction: What have we learned?. Current Opinion in Behavioral Sciences.

[CR33] Hales CA, Houghton CJ, Robinson ES (2017). Behavioural and computational methods reveal differential effects for how delayed and rapid onset antidepressants effect decision making in rats. European Neuropsychopharmacology.

[CR34] Hales CA, Robinson ES, Houghton CJ (2016). Diffusion modelling reveals the decision making processes underlying negative judgement bias in rats. PloS One.

[CR35] Harding EJ, Paul ES, Mendl M (2004). Animal behaviour: Cognitive bias and affective state. Nature.

[CR36] Herrnstein RJ (1961). Relative and absolute strength of response as a function of frequency of reinforcement. Journal of the Experimental Analysis of Behavior.

[CR37] Hisey, E. E., Fritsch, E. L., Newman, E. L., Ressler, K. J., Kangas, B. D., & Carlezon Jr, W. A, (2023). Early life stress in male mice blunts responsiveness in a translationally-relevant reward task. *Neuropsychopharmacology*10.1038/s41386-023-01610-7 PMC10579416 37258714

[CR38] Hodgkin AL, Huxley AF (1952). A quantitative description of membrane current and its application to conduction and excitation in nerve. The Journal of Physiology.

[CR39] Huys QJ, Eshel N, O’Nions E, Sheridan L, Dayan P, Roiser JP (2012). Bonsai trees in your head: How the pavlovian system sculpts goal-directed choices by pruning decision trees. PLoS Computational Biology.

[CR40] Huys QJ, Guitart-Masip M, Dolan RJ, Dayan P (2015). Decision-theoretic psychiatry. Clinical. Psychological Science.

[CR41] Huys QJ, Maia TV, Frank MJ (2016). Computational psychiatry as a bridge from neuroscience to clinical applications. Nature Neuroscience.

[CR42] Huys QJ, Pizzagalli DA, Bogdan R, Dayan P (2013). Mapping anhedonia onto reinforcement learning: A behavioural meta-analysis. Biology of Mood & Anxiety Disorders.

[CR43] Iigaya K, Jolivald A, Jitkrittum W, Gilchrist ID, Dayan P, Paul E, Mendl M (2016). Cognitive bias in ambiguity judgements: Using computational models to dissect the effects of mild mood manipulation in humans. PloS One.

[CR44] Insel T, Cuthbert B, Garvey M, Heinssen R, Pine DS, Quinn K, Sanislow C, Wang P (2010). Research domain criteria (rdoc): Toward a new classification framework for research on mental disorders. American Journal of Psychiatry.

[CR45] Ironside M, Kumar P, Kang M-S, Pizzagalli DA (2018). Brain mechanisms mediating effects of stress on reward sensitivity. Current Opinion in Behavioral Sciences.

[CR46] Jolles JW (2021). Broad-scale applications of the raspberry pi: A review and guide for biologists. Methods in Ecology and Evolution.

[CR47] Jones S, Neville V, Higgs L, Paul ES, Dayan P, Robinson ES, Mendl M (2018). Assessing animal affect: An automated and self-initiated judgement bias task based on natural investigative behaviour. Scientific Reports.

[CR48] Kangas BD, Wooldridge LM, Luc OT, Bergman J, Pizzagalli DA (2020). Empirical validation of a touchscreen probabilistic reward task in rats Translational. Psychiatry.

[CR49] Kangas BD, Der-Avakian A, Pizzagalli DA (2022). Probabilistic reinforcement learning and anhedonia Curr Top. Behav Neurosci.

[CR50] Kremer L, Holkenborg SK, Reimert I, Bolhuis J, Webb L (2020). The nuts and bolts of animal emotion. Neuroscience & Biobehavioral Reviews.

[CR51] Kumar V, Bhat ZA, Kumar D (2013). Animal models of anxiety: A comprehensive review. Journal of Pharmacological and Toxicological Methods.

[CR52] Lagisz, M., Zidar, J., Nakagawa, S., Neville, V., Sorato, E., Paul, E. S., Bateson, M., Mendl, M., & Løvlie, H. (2020). Optimism, pessimism and judgement bias in animals: A systematic review and meta-analysis. *Neuroscience & Biobehavioral Reviews*10.1016/j.neubiorev.2020.07.012 32682742

[CR53] LeDoux J (2012). Rethinking the emotional brain. Neuron.

[CR54] Loewenstein G (2000). Emotions in economic theory and economic behavior. American economic review.

[CR55] Loosen AM, Hauser TU (2020). Towards a computational psychiatry of juvenile obsessive-compulsive disorder. Neuroscience & Biobehavioral Reviews.

[CR56] Ma WJ, Jazayeri M (2014). Neural coding of uncertainty and probability. Annual Review of Neuroscience.

[CR57] Mendl M, Burman OH, Parker RM, Paul ES (2009). Cognitive bias as an indicator of animal emotion and welfare: Emerging evidence and underlying mechanisms. Applied Animal Behaviour Science.

[CR58] Mendl M, Burman OH, Paul ES (2010). An integrative and functional framework for the study of animal emotion and mood. Proceedings of the Royal Society B: Biological Sciences.

[CR59] Mendl M, Neville V, Paul ES (2022). Bridging the gap: Human emotions and animal emotions. Affective Science.

[CR60] Mendl M, Paul ES (2020). Animal affect and decision-making. Neuroscience and Biobehavioral Reviews.

[CR61] Meyniel F, Goodwin GM, Deakin JW, Klinge C, MacFadyen C, Milligan H, Mullings E, Pessiglione M, Gaillard R (2016). A specific role for serotonin in overcoming effort cost. Elife.

[CR62] Millner, A. J., den Ouden, H. E., Gershman, S. J., Glenn, C. R., Kearns, J. C., Bornstein, A. M., Marx, B. P., Keane, T. M., & Nock, M. K. (2019). Suicidal thoughts and behaviors are associated with an increased decision-making bias for active responses to escape aversive states. *Journal of Abnormal Psychology,**128*(2), 106. 10.1037/abn0000395 30589305

[CR63] Mobbs D, Adolphs R, Fanselow MS, Barrett LF, LeDoux JE, Ressler K, Tye KM (2019). Viewpoints: Approaches to defining and investigating fear. Nature Neuroscience.

[CR64] Montague PR, Dayan P, Sejnowski TJ (1996). A framework for mesencephalic dopamine systems based on predictive hebbian learning. Journal of Neuroscience.

[CR65] Montague PR, Dolan RJ, Friston KJ, Dayan P (2012). Computational psychiatry. Trends in Cognitive Sciences.

[CR66] Moutoussis M, Story G, Dolan RJ (2015). The computational psychiatry of reward: Broken brains or misguided minds?. Frontiers in Psychology.

[CR67] Nesse RM (2000). Is depression an adaptation?. Archives of general psychiatry.

[CR68] Nettle D, Bateson M (2012). The evolutionary origins of mood and its disorders. Current Biology.

[CR69] Neville V, Dayan P, Gilchrist ID, Paul ES, Mendl M (2021). Dissecting the links between reward and loss, decision-making, and self-reported affect using a computational approach. PLOS Computational Biology.

[CR70] Neville V, Dayan P, Gilchrist ID, Paul ES, Mendl M (2021). Dissecting the links between reward and loss, decision-making, and self-reported affect using a computational approach. PLOS Computational Biology.

[CR71] Neville V, Dayan P, Gilchrist ID, Paul ES, Mendl M (2021). Using primary reinforcement to enhance translatability of a human affect and decision-making judgment bias task. Journal of Cognitive Neuroscience.

[CR72] Neville, V., King, J., Gilchrist, I. D., Dayan, P., Paul, E. S., & Mendl, M. (2020). Reward and punisher experience alter rodent decision-making in a judgement bias task. *Scientific Reports,**10*(1), 1– 14. 10.1038/s41598-020-68737-1 PMC7366639 32678247

[CR73] Neville V, Nakagawa S, Zidar J, Paul ES, Lagisz M, Bateson M, Løvlie H, Mendl M (2020). Pharmacological manipulations of judgement bias: A systematic review and meta-analysis. Neuroscience and Biobehavioral Reviews.

[CR74] Noworyta-Sokolowska K, Kozub A, Jablonska J, Rodriguez Parkitna J, Drozd R, Rygula R (2019). Sensitivity to negative and positive feedback as a stable and enduring behavioural trait in rats. Psychopharmacology.

[CR75] Otto AR, Eichstaedt JC (2018). Real-world unexpected outcomes predict city-level mood states and risk-taking behavior. PloS one.

[CR76] Ousdal OT, Huys Q, Mildë AM, Craven AR, Ersland L, Endestad T, Melinder A, Hugdahl K, Dolan RJ (2018). The impact of traumatic stress on pavlovian biases. Psychological medicine.

[CR77] Overstreet DH, Friedman E, Mathé AA, Yadid G (2005). The flinders sensitive line rat: A selectively bred putative animal model of depression. Neuroscience & Biobehavioral Reviews.

[CR78] Panksepp J (2005). Affective consciousness: Core emotional feelings in animals and humans. Consciousness and Cognition.

[CR79] Panksepp J (2011). The basic emotional circuits of mammalian brains: do animals have affective lives?. Neuroscience & Biobehavioral Reviews.

[CR80] Paul ES, Harding EJ, Mendl M (2005). Measuring emotional processes in animals: The utility of a cognitive approach. Neuroscience & Biobehavioral Reviews.

[CR81] Paul ES, Sher S, Tamietto M, Winkielman P, Mendl MT (2020). Towards a comparative science of emotion: Affect and consciousness in humans and animals. Neuroscience & Biobehavioral Reviews.

[CR82] Pike, A. C. & Robinson, O. J. (2022). Reinforcement learning in patients with mood and anxiety disorders vs control individuals: A systematic review and meta-analysis. *JAMA psychiatry*. 10.1001/jamapsychiatry.2022.0051 PMC8892374 35234834

[CR83] Piray, P., Dezfouli, A., Heskes, T., Frank, M. J., & Daw, N. D. (2019). Hierarchical bayesian inference for concurrent model fitting and comparison for group studies. *PLoS Computational Biology*, 15(6) 10.1371/journal.pcbi.1007043 PMC6581260 31211783

[CR84] Poirier C, Bateson M, Gualtieri F, Armstrong EA, Laws GC, Boswell T, Smulders TV (2019). Validation of hippocampal biomarkers of cumulative affective experience. Neuroscience & Biobehavioral Reviews.

[CR85] Rae CL, Critchley HD, Seth AK (2019). A bayesian account of the sensory-motor interactions underlying symptoms of tourette syndrome. Frontiers in Psychiatry.

[CR86] Ratcliff R (1978). A theory of memory retrieval. Psychological review.

[CR87] Rescorla RA, Wagner AR (1972). A theory of pavlovian conditioning: Variations in the effectiveness of reinforcement and nonreinforcement. Classical conditioning II: Current research and Theory.

[CR88] Rivalan M, Valton V, Series P, Marchand AR, Dellu-Hagedorn F (2013). Elucidating poor decision-making in a rat gambling task. PLoS One.

[CR89] Rolls ET (2013). What are emotional states, and why do we have them?. Emotion Review.

[CR90] Royce JR (1977). On the construct validity of open-field measures. Psychological bulletin.

[CR91] Ruhé HG, Mason NS, Schene AH (2007). Mood is indirectly related to serotonin, norepinephrine and dopamine levels in humans: a meta-analysis of monoamine depletion studies. Molecular psychiatry.

[CR92] Rupniak N (2003). Animal models of depression: Challenges from a drug development perspective. Behavioural Pharmacology.

[CR93] Rupprechter S, Stankevicius A, Huys QJ, Steele JD, Seriès P (2018). Major depression impairs the use of reward values for decision-making. Scientific reports.

[CR94] Rutledge RB, Skandali N, Dayan P, Dolan RJ (2014). A computational and neural model of momentary subjective well-being. Proceedings of the National Academy of Sciences.

[CR95] Saez I, Gu X (2023). Invasive computational psychiatry. Biological psychiatry.

[CR96] Schrijver NC, Bahr NI, Weiss IC, Würbel H (2002). Dissociable effects of isolation rearing and environmental enrichment on exploration, spatial learning and hpa activity in adult rats. Pharmacology Biochemistry and Behavior.

[CR97] Schüller T, Fischer AG, Gruendler TO, Baldermann JC, Huys D, Ullsperger M, Kuhn J (2020). Decreased transfer of value to action in tourette syndrome. Cortex.

[CR98] Schultz W, Apicella P, Ljungberg T (1993). Responses of monkey dopamine neurons to reward and conditioned stimuli during successive steps of learning a delayed response task. Journal of neuroscience.

[CR99] Schultz W, Dayan P, Montague PR (1997). A neural substrate of prediction and reward. Science.

[CR100] Series, P. (2020). Computational psychiatry: A primer. MIT Press.

[CR101] Slattery DA, Markou A, Cryan JF (2007). Evaluation of reward processes in an animal model of depression. Psychopharmacology.

[CR102] Spiegler KM, Palmieri J, Pang KC, Myers CE (2020). A reinforcement-learning model of active avoidance behavior: Differences between sprague dawley and wistar-kyoto rats. Behavioural Brain Research.

[CR103] Stephan KE, Bach DR, Fletcher PC, Flint J, Frank MJ, Friston KJ, Heinz A, Huys QJ, Owen MJ, Binder EB (2016). Charting the landscape of priority problems in psychiatry, part 1: classification and diagnosis. The Lancet Psychiatry.

[CR104] Stephan KE, Mathys C (2014). Computational approaches to psychiatry. Current Opinion in Neurobiology.

[CR105] Sutton RS, Barto AG (1981). Toward a modern theory of adaptive networks: Expectation and prediction. Psychological Review.

[CR106] Sutton, R. S. & Barto, A. G. (2018). Introduction to reinforcement learning. MIT press Cambridge, 2 edition.

[CR107] Swanson K, Averbeck BB, Laubach M (2022). Noradrenergic regulation of two-armed bandit performance. Behavioral Neuroscience.

[CR108] Theisen M, Lerche V, von Krause M, Voss A (2021). Age differences in diffusion model parameters: A meta-analysis. Psychological Research.

[CR109] Ulrichsen KM, Alnaes D, Kolskar KK, Richard G, Sanders A-M, Dorum ES, Ihle-Hansen H, Pedersen ML, Tornas S, Nordvik JE, Westlye LT (2020). Dissecting the cognitive phenotype of post-stroke fatigue using computerized assessment and computational modeling of sustained attention. Psychological research.

[CR110] Valletta JJ, Torney C, Kings M, Thornton A, Madden J (2017). Applications of machine learning in animal behaviour studies. Animal Behaviour.

[CR111] Valton V, Romaniuk L, Steele JD, Lawrie S, Seriès P (2017). Comprehensive review: Computational modelling of schizophrenia. Neuroscience & Biobehavioral Reviews.

[CR112] van Ravenzwaaij, D., Dutilh, G., & Wagenmakers, E.-J. (2012). A diffusion model decomposition of the effects of alcohol on perceptual decision making. *Psychopharmacology,**218*, 1017–1025. 10.1007/s00213-011-2435-9 PMC3266508 21842158

[CR113] Vinckier, F., Jaffre, C., Gauthier, C., Smajda, S., Abdel-Ahad, P., Le Bouc, R., Daunizeau, J., Fefeu, M., Borderies, N., Plaze, M., et al. (2022). Elevated effort cost identified by computational modeling as a distinctive feature explaining multiple behaviors in patients with depression. *Biological Psychiatry: Cognitive Neuroscience and Neuroimaging,**7*(11), 1158–1169. 10.1016/j.bpsc.2022.07.011 35952972

[CR114] Vulkan N (2000). An economist’s perspective on probability matching. Journal of Economic Surveys.

[CR115] Wallace J (2000). Humane endpoints and cancer research Institute for Laboratory. Animal Research.

[CR116] Whitton AE, Treadway MT, Pizzagalli DA (2015). Reward processing dysfunction in major depression, bipolar disorder and schizophrenia. Current Opinion in Psychiatry.

[CR117] Widrow, B. & Hoff, M. E. (1960). Adaptive switching circuits. Technical report, Stanford Univ Ca Stanford Electronics Labs.

[CR118] Willner P (2017). The chronic mild stress (cms) model of depression: History, evaluation and usage. Neurobiology of Stress.

[CR119] Willner P, Towell A, Sampson D, Sophokleous S, Muscat R (1987). Reduction of sucrose preference by chronic unpredictable mild stress, and its restoration by a tricyclic antidepressant. Psychopharmacology.

[CR120] Wilson, R. C. & Collins, A. G. (2019). Ten simple rules for the computational modeling of behavioral data. *eLife*, 8, e49547 10.7554/eLife.49547 PMC6879303 31769410

[CR121] Wooldridge LM, Bergman J, Pizzagalli DA, Kangas BD (2021). Translational assessments of reward responsiveness in the marmoset. International Journal of Neuropsychopharmacology.

